# Single-cell transcriptome and TCR profiling reveal activated and expanded T cell populations in Parkinson’s disease

**DOI:** 10.1038/s41421-021-00280-3

**Published:** 2021-07-20

**Authors:** Pingping Wang, Lifen Yao, Meng Luo, Wenyang Zhou, Xiyun Jin, Zhaochun Xu, Shi Yan, Yiqun Li, Chang Xu, Rui Cheng, Yan Huang, Xiaoyu Lin, Kexin Ma, Huimin Cao, Hongxin Liu, Guangfu Xue, Fang Han, Huan Nie, Qinghua Jiang

**Affiliations:** 1 School of Life Science and Technology, Harbin Institute of Technology, Harbin, Heilongjiang China; 2 Department of Neurology, First Affiliated Hospital of Harbin Medical University, Harbin, Heilongjiang China; 3 Key Laboratory of Biological Big Data (Harbin Institute of Technology), Ministry of Education, Harbin, Heilongjiang China

**Keywords:** Bioinformatics, Autoimmunity

## Abstract

Given the chronic inflammatory nature of Parkinson’s disease (PD), T cell immunity may be important for disease onset. Here, we performed single-cell transcriptome and TCR sequencing, and conducted integrative analyses to decode composition, function and lineage relationship of T cells in the blood and cerebrospinal fluid of PD. Combined expression and TCR-based lineage tracking, we discovered a large population of CD8^+^ T cells showing continuous progression from central memory to terminal effector T cells in PD patients. Additionally, we identified a group of cytotoxic CD4^+^ T cells (CD4 CTLs) remarkably expanded in PD patients, which derived from Th1 cells by TCR-based fate decision. Finally, we screened putative TCR–antigen pairs that existed in both blood and cerebrospinal fluid of PD patients to provide potential evidence for peripheral T cells to participate in neuronal degeneration. Our study provides valuable insights and rich resources for understanding the adaptive immune response in PD.

## Introduction

Parkinson’s disease (PD) is the second most common neurodegenerative disorder in the aging population after Alzheimer’s disease. PD is characterized by the loss of dopaminergic neurons in substantia nigra, leading to severe and progressive dyskinesia, including bradykinesia, rest tremor, rigidity and a variety of non-motor symptoms, such as disorders of mood, affect with apathy, and cognitive dysfunction^1^. It is estimated that PD affects one percent of the population over the age of 60 years^2,3^. Overall, more than 10 million people worldwide have PD^4^, and 80% of PD patients will eventually develop dementia^5^.

Increasing studies suggest that immune system dysfunction plays important roles in the pathogenesis of PD, including clinical and genetic associations with autoimmune disease, cellular and humoral immune dysfunction, imaging evidence of inflammatory cell activation and immunomodulatory disorders in experimental models of PD^6–9^. This complex disease is likely of autoimmune origin, but many questions remain unanswered despite a vast amount of available literature. On the one hand, several studies have reported the alteration of the percentage of peripheral blood T cells in PD patients^10^, but the relative contribution of each cell subtype to the disease etiology remains unclear^10^. On the other hand, CD8^+^ and CD4^+^ T cells were reported to invade the brain in both postmortem human PD specimens and in the mouse model of PD^9,11^, but the composition and interaction of T cell subtypes in human peripheral blood and cerebrospinal fluid and their potential ability to infiltrate the central nervous system remain unclear.

Single-cell RNA sequencing has emerged as a powerful technology for studying the heterogeneity of complex tissues, which provides higher resolution of cellular differences and reveals important functional insights that are masked in bulk analysis of cell populations^12,13^. Single-cell T cell receptor (TCR) sequencing provides TCR sequences for each cell^14^. The same TCR sequences indicate T cell clonal expansion patterns and T cell lineages, which are pivotal for recognizing endogenous and exogenous antigens presented by the major histocompatibility complex (MHC)^15^. Recently, single-cell transcriptome and TCR sequencing has been applied to analyze immune cells in patients with Alzheimer’s disease and multiple sclerosis, revealing T cell expansion signatures and their relationship with nervous system inflammation^16,17^. Large-scale single-cell sequencing of lymphocytes may help us to better understand the adaptive immune response in PD.

Given the chronic inflammatory nature of PD, T cell immunity may be important for disease onset. Here, we used single-cell transcriptome and TCR sequencing to systematically characterize the composition, function and lineage relationship of T lymphocytes in the blood and cerebrospinal fluid (CSF) of PD. In total, 21 T cell subsets with distinct functions were identified from 103,365 T cells. Integrative analyses of single-cell gene expression and TCRs revealed connectivity and potential differentiation trajectories of these subtypes and provided novel evidence of clonal expansion of T lymphocytes patrolling in the blood and cerebrospinal fluid of PD. This unprecedentedly large-scale transcriptome and immune profiling data of T cells can be used as a valuable resource for studying the basic characteristics of PD and potentially guiding effective immunotherapy strategies.

## Results

### Single-cell transcriptome and TCR sequencing of T cells in PD patients and healthy controls

**Fig. 1:**
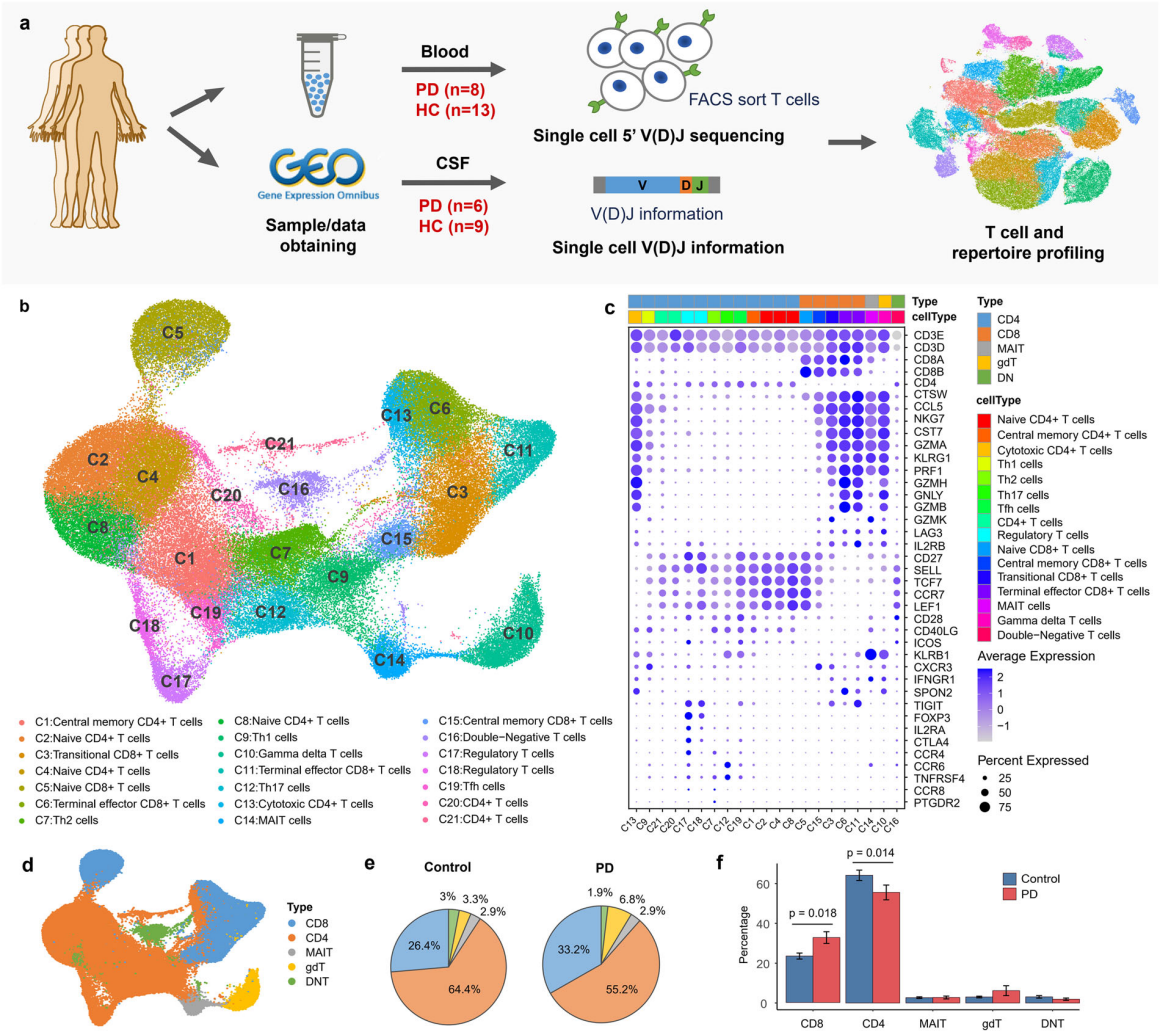
Single-cell transcriptome profiling of T cells. **a** Overview of experimental design. CD3^+^ T cells from the blood of 8 PD patients and 6 healthy controls were sorted by FACS and simultaneously subject to single-cell transcriptome and immune sequencing with 5’ V(D)J capture. Single-cell TCR data from cerebrospinal fluid were downloaded from GEO with accession ID GSE134578. Batch effect correction and unsupervised clustering were performed after merging single-cell datasets. **b** UMAP projection of 103,365 single T cells, showing 21 clusters. Each dot corresponds to one cell and is colored according to cell cluster. **c** Dot plot shows the expression of marker genes for 21 cell clusters. The size of the dot corresponds to the percentage of cells expressing the gene in each cluster, and the color represents the average log normalized gene expression. Markers were ordered to visualize the differences between cell types. **d** UMAP projection of T cells colored by the 5 major T cell types, including CD8^+^ T cells (CD8), CD4^+^ T cells (CD4), mucosal-associated invariant T cells (MAIT), gamma delta T cells (gdT) and double-negative T cells (DNT). **e** Pie charts showing the percentages of the five major T cell types in the blood of PD patients and healthy controls. For each cell type, the percentage was obtained by dividing the number of cells in that cell type by the total number of cells in all PD patients or in all healthy controls. **f** Barplot showing the percentages for different T cell types identified from single cell analysis. Error bars represent standard error of the mean.

We conducted a comprehensive analysis of single-cell transcriptome and TCR profiling of T cells in the blood and cerebrospinal fluid of PD patients (Fig. 1a). Fresh blood samples were collected from 8 PD patients and 6 healthy controls. CD3^+^ T cells were sorted by flow cytometry, and single-cell 5’ gene-expression and V(D)J libraries were prepared on the 10× platform (10× Genomics, CA, USA). Another 7 single-cell datasets from healthy controls were downloaded from publicly available datasets (Supplementary Table S1). In addition, publicly available single-cell immune profiling datasets from CSF, including 6 PD patients and 9 healthy controls^16^, were compared to better understand clonal expansion of lymphocyte T cells in PD. In total, we obtained single-cell transcriptome data for 103,365 T cells and single-cell TCR sequencing data for 113,690 T cells, of which 84,384 cells have both gene expression and TCR profiling data (Supplementary Table S1).

### T cells exhibit a specific composition and transcriptome in PD

To reveal the internal structure and potential functional subtypes of the entire T cell population, we used a graph-based clustering approach implemented in Seurat^18,19^ to perform unsupervised clustering of all T cells. T cells were visualized in 2D space using uniform manifold approximation and projection (UMAP) based on the gene expression profiling. In total, we identified 21 distinct clusters representing different cell types, including 11 clusters for conventional CD4^+^ T cells, 2 clusters for regulatory CD4^+^ T cells, 5 clusters for CD8^+^ T cells, 1 gamma delta T cell cluster, 1 MAIT cell cluster and 1 double-negative T cell cluster (Fig. 1b). Cell types were manually annotated by assessing the expression of classic marker genes and their expression similarity with purified bulk RNA-seq datasets^20–24^ (Fig. 1c; Supplementary Fig. S1). Five major cell types, including CD8^+^ T cells (CD8), CD4^+^ T cells (CD4), mucosal associated invariant T cells (MAIT), gamma delta T cells (gdT) and double-negative T cells (DNT), were highlighted in Fig. 1d.

Regarding CD4^+^ T cells, 3 clusters (C2, C4 and C8 clusters) were annotated as naïve CD4^+^ T cells characterized by naïve T cell markers SELL, CCR7, TCF7 and LEF1; C1, C9, C7, C12 and C19 were separately annotated as central memory CD4^+^ T cells, classic Th1, Th2, Th17 and Tfh cells, respectively, based on correlation analysis with bulk RNA-seq datasets of purified immune cells^20–24^ (Supplementary Fig. S1a); C13 was annotated as cytotoxic CD4^+^ T cells (CD4 CTL) with high expression of CD4 and cytotoxic genes GZMA, GZMB, PRF1 and NKG7; C17 and C18 were annotated as regulatory CD4^+^ T cells with high expression of Treg markers FOXP3, CTLA4, TIGIT and IL2RA; C20 and C21 were not assigned to specific CD4^+^ T cell types due to insufficient evidence (Fig. 1c; Supplementary Fig. S1).

For CD8^+^ T cells, the C5 cluster was annotated as naïve CD8^+^ T cells that highly expressed naïve cell markers SELL, CCR7, TCF7 and LEF1; 2 clusters (C6 and C11) were annotated as terminal effector CD8^+^ T cells characterized by effector markers, such as GZMA, GZMB, PRF1, NKG7; C3 cluster was annotated as transitional CD8^+^ T cells with high expression of the transitional marker gene GZMK^25^; the C15 cluster was annotated as central memory CD8^+^ T cells (T_CM_) with high expression of T_CM_ markers CD27, SELL and CCR7 (Fig. 1c; Supplementary Fig. S1).

The remaining T cells formed 3 clusters, including 1 gamma delta T cell cluster, 1 MAIT cell cluster and 1 double-negative T cell cluster. C10 was annotated as Vd2 gamma delta T cells, in which 93% of the cells exhibited high gene expression of TRDV2 and TRGV9 and αβTCR was not detected in 93% of the cells (Supplementary Table S2). In total, 69% of the cells from C10 were annotated as Vd2 gd T cells by purified bulk RNA-seq datasets Monaco et al.^21^ (Supplementary Fig. S1). C16 was annotated as double-negative T cells, in which more than 60% of the cells express neither CD4 nor CD8 (Supplementary Fig. S1). C14 was annotated as MAIT cells with absolute superiority of the recombination ratio of TRAV1-2 and TRAJ33 gene segments in the TCRα chain; moreover, correlation analysis also revealed the closest similarity to purified MAIT cells from Monaco et al.^21^ (Fig. 1c; Supplementary Table S2 and Fig. S1a).

To understand whether PD patients follow the reported T lymphocyte changes^10^, we compared the proportion of CD4^+^ T cells and CD8^+^ T cells in the blood between PD patients and healthy controls. Among the identified cell types in our single-cell transcriptome analysis, the proportion of CD8^+^ T cells was significantly increased in the blood of PD patients compared to healthy controls (*t*-test, *P*-value = 0.018), whereas the proportion of CD4^+^ T cells significantly decreased (*t*-test, *P*-value = 0.014) (Fig. 1e, f). The overall CD4/CD8 ratio in PD patients (ratio = 1.66) was significantly reduced compared with healthy controls (ratio = 2.44) (*t*-test, *P-*value = 0.0048). Published studies have shown that the CD4/CD8 ratio in the peripheral blood of healthy adults is approximately 2:1, and an altered ratio is indicative of diseases that are associated with the immunodeficiency or autoimmunity^26–28^. Significant decrease in the CD4/CD8 ratio may indicate an immune disorder in PD.

### Clonally expanded T cells in the blood and CSF of PD

**Fig. 2:**
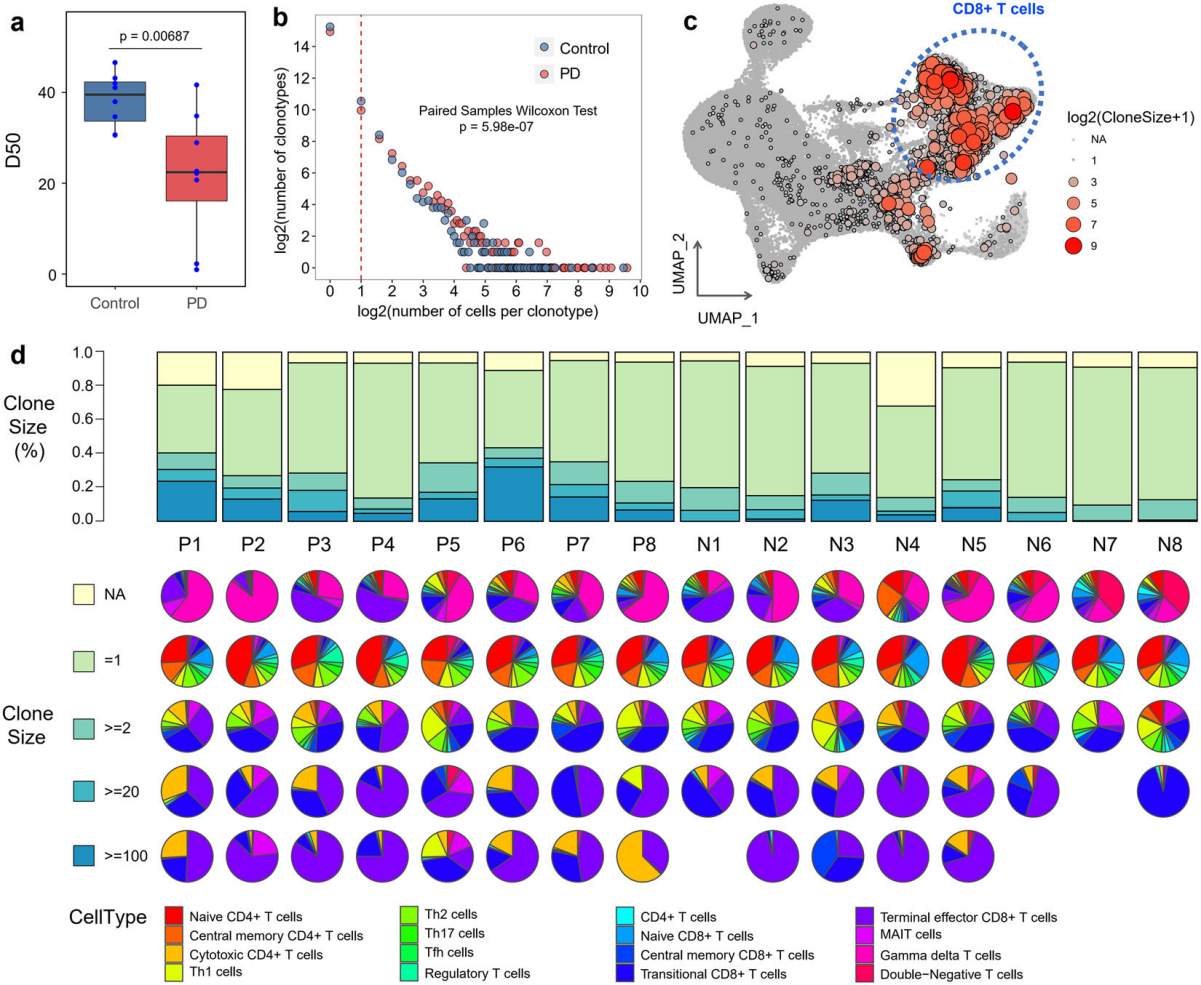
Single-cell immune profiling of T cells. **a** Blood TCR diversity comparison between PD patients and healthy controls. TCR diversity was measured by D50, which is proven robust for the sequencing library size. *P* value was estimated using two-sided Wilcoxon test. **b** The association between the number of T cell clonotypes and the number of cells per clonotype. Downsampling was used to avoid bias caused by the total number of detected T cells between PD patients and healthy controls. The dashed line separates nonclonal and clonal cells, with the latter identified by repeated usage of αβTCRs. *P* value was estimated using paired samples Wilcoxon test. **c** UMAP plot showing the distribution of clonally expanded T cells. Each dot represents a unique clonotype in a T cell cluster, and the coordinates are the average coordinates of the cells belonging to this clonotype. The color and size of the dot both reflect the clone size in each cluster. **d** Clonal composition of T cells in samples. The top panel shows the distribution of clonotypes by size (NA, = 1, ≥2, ≥20 and ≥100 cells, NA represents cells with no αβTCR sequence detected). The bottom pie charts show the cell type composition of clonotypes from each sample stratified by clone size.

To gain insight into the clonal expansion of T cells in PD, we performed comparison analysis for scTCR-seq data from PD patients and healthy controls. Cells with the same CDR3 sequences for both the TCR α-chain and β-chain were defined as the same clonotype. We detected 113,690 cells from single-cell TCR sequencing data, forming 87,832 unique clonotypes, in which 4458 clonotypes contained at least two cells, indicating the clonal expansion of T cells (Supplementary Table S5). T cell diversity in the blood was significantly lower in PD patients compared with healthy controls (*t*-test, *P-*value = 6.87E−3, Fig. 2a). The number of clonotypes with the same clone size was significantly increased in PD patients compared with healthy controls (100 random sampling tests, median *P-*value = 5.98e−7, Fig. 2b). These results indicate the existence of T cell clonal expansion in the blood of PD patients.

In CSF, T cell diversity was slightly reduced in PD patients compared with healthy controls, and the statistical *P-*value was not significant, which may be due to the small number of detected cells and the small number of samples (*t*-test, *P*-value = 0.076, Supplementary Fig. S2a). However, the number of clonotypes with the same clone size was significantly increased in the CSF of PD patients compared with healthy controls (100 random sampling tests, median *P-*value = 6.25e−3, Supplementary Fig. S2b). And, the percentage of T cells with clone size > 2 was significantly increased in the CSF of PD patients compared to healthy controls (*t*-test, *P-*value = 0.033, Supplementary Fig. S2c). These results suggest that T cell clonal expansion also occurs in CSF of PD patients.

Clonally expanded T cells were widely distributed in each cluster, especially in CD8^+^ T cells (Fig. 2c). T cell composition distributed by clone size (NA, = 1, ≥2, ≥20, ≥100, NA means no TCR detected in these cells) in each blood sample is shown in Fig. 2d. The percentage of T cells with clone size ≥2 and ≥100 were significantly increased in the blood of PD patients compared with healthy controls (*t*-test, *P* value = 0.0030 and 0.0074, respectively) (Fig. 2d). We observed that cell type composition in each sample varied by clone size (Fig. 2d). T cells without αβTCR detected in scTCR-seq were mainly Vd2 gd T cells, while the clonotypes containing only one cell were mainly naïve CD4^+^ T cells (Fig. 2d). Larger clonotypes exhibited a nonuniform distribution of cell types with an enrichment for transitional and terminal effector CD8^+^ T cells (Fig. 2d).

### Clonal linkage of CD8^+^ T cells form a gradient of transcriptional states in PD

**Fig. 3:**
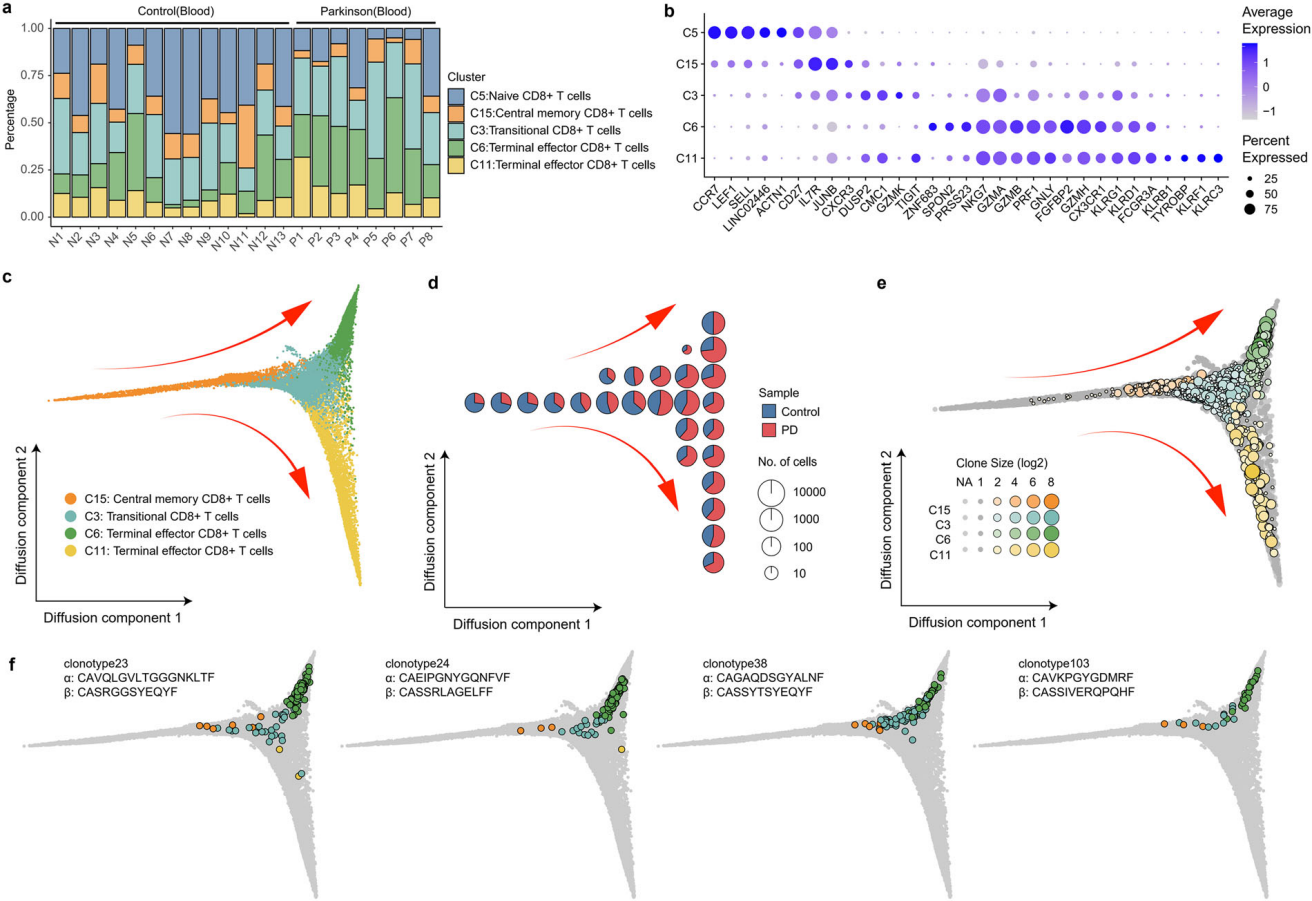
Relationship among CD8^+^ T cell clusters based on both transcriptome and TCR data. **a** Percentage of CD8^+^ T cells in each sample shows reduction of naïve CD8^+^ T cells (C5 cluster) and expansion of terminal effector CD8^+^ T cells (C6 cluster) in the blood of PD patients. **b** Dot plot shows the expression of the Top 5 highly expressed genes in each cluster. The size of the dot corresponds to the percentage of cells expressing the gene in each cluster, and the color represents the average log normalized gene expression. **c** Pseudotime ordering of CD8^+^ T cells in a diffusion trajectory using the first two diffusion components. Each dot represents a cell colored by different cell cluster. The main trajectories were indicated with arrows. **d** Cell sample composition in the diffusion trajectories. The diffusion trajectory in **c** was divided into 10 × 10 square grids according to the horizontal and vertical coordinates, and each pie chart reflects the sample composition of cells in each grid area. **e** Clonotypes distributed in the diffusion trajectories. Each dot represents a unique clonotype in each cluster. The size of the dot reflects the clone size in each cluster. Clonotypes with clone size >1 were colored by cluster. Nonclonal clonotypes are colored in gray. The coordinates of each clonotype were calculated by the average coordinates of the cells in the clonotype. **f** Examples of clonally expanded CD8^+^ T cells in different cell types. Cells from 4 clonotypes (clonotype23, clonotype24, clonotype38 and clonotype103) were highlighted in the diffusion trajectories. Each dot represents a cell colored based on its cluster.

We performed in-depth analysis of CD8^+^ T cells across all PD patients and healthy controls. Interestingly, CD8^+^ T cells exhibited a nonuniform distribution of functional states with significant enrichment for terminal effector CD8^+^ T cells (C6 cluster, *t*-test, FDR = 0.015) and depletion of naïve CD8^+^ T cells (C5 cluster, *t*-test, FDR = 0.012) in the blood of PD patients (Fig. 3a). The expression of signature genes fluctuated significantly in these five CD8^+^ T cell clusters, and terminal effector CD8^+^ T cells exhibited wider and higher expression of cytotoxic genes (Fig. 3b). Fisher’s exact test showed that clonally expanded T cells in PD patients were significantly enriched in transitional and terminal effector CD8^+^ T cells, especially in C3 and C6 clusters (Fisher’s exact test, FDR = 1.10e-46 and 1.02e-8, respectively).

To further understand the relationships among CD8^+^ T cell clusters, we used diffusion maps to visualize these cells on a pseudotime trajectory (Fig. 3c). Interesting, the first diffusion component separated central memory cells from activated CD8^+^ T cells and was highly correlated with cytotoxic-related genes, such as GZMH, PRF1, FGFBP2, as well as proteins regulating cell migration and adhesion, such as CX3CR1, and ADGRG1 (Fig. 3c; Supplementary Fig. S3a–b). The second diffusion component showed two different differentiation directions of terminal effector CD8^+^ T cells (Fig. 3c). The upper branch (C6 cluster) was highly correlated with cell adhesion proteins, such as ITGAM and ITGB1, and the tissue-resident T cell transcription regulator protein ZNF683 (encodes for Hobit), whereas the lower differentiation branch (C11 cluster) was highly correlated with killer-like receptors, such as KLRC3 and KLRF1, and killer cell immunoglobulin-like receptors, such as KIR2DL3 and KIR3DL2 (Fig. 3c; Supplementary Fig. S3c, d).

To further understand the functional differences between terminal effector CD8^+^ T cells, we analyzed differentially expressed genes between the upper and lower differentiation branches (C6 and C11 clusters) (Supplementary Fig. S3e). The upper branch (C6 cluster) highly expressed cell adhesion and migration genes, such as ITGAM, ITGB1, CD226 and S100A4; T-cell activation and proliferation markers, such as CD52 and S100A6; and tissue-resident T cell transcription regulator protein ZNF683 (Hobit) (Supplementary Fig. S3e). The KEGG pathway analysis results revealed that the C6 up-regulated genes were highly associated with cell adhesion molecules (KEGG: hsa04514, FDR = 0.0015) and leukocyte transendothelial migration (KEGG: hsa04670, FDR = 0.0047) (Supplementary Fig. S3f), suggesting that these cells may be involved in tissue immunity. Genes related to cell survival and cytotoxic function, such as PRSS23^29^, SPON2^30^ and ZNF683 (Hobit)^31,32^, were also highly expressed in the C6 cluster (Fig. 3b; Supplementary Fig. S3e). The lower branch (C11 cluster) highly expressed genes enriched in the natural killer cell-mediated cytotoxicity pathway (KEGG: hsa04650, FDR = 2.78e−10, Supplementary Fig. S3g), including killer-like receptors KLRC3, KLRF1, and KLRB1 and killer cell immunoglobulin-like receptors KIR3DL1, KIR3DL2 and KIR2DL3 (Supplementary Fig. S3e). This group of CD8^+^ T cells functioned more like nonclassical NKT cells^33^.

Moreover, the sample composition distribution of the cells in diffusion trajectory reveals that the proportion of CD8^+^ T cells in the blood of PD patients gradually increased with the process of differentiation, especially in the upper differentiation branch (Fig. 3d). Larger clonotypes tend to be located at the end of the effector branch (Fig. 3e). A process of transformation from central memory CD8^+^ T cells (C15 cluster) to transitional CD8^+^ T cells (C3 cluster) followed by terminal effector CD8^+^ T cells (C6 cluster) in the blood of PD patients (Fig. 3d) is clearly observed. The distribution of T cell clonotypes sharing the same TCRs further supported this transformation (Fig. 3f). Tracking T cell clonotypes and transcriptional phenotypes, we found that 55 clonotypes contained cells distributed in central memory CD8^+^ T cells (C15 cluster), transitional CD8^+^ T cells (C3 cluster), and terminal effector CD8^+^ T cells (C6 cluster), such as clonotype23, clonotype24, clonotype38 and clonotype103 (Fig. 3f), suggesting that TCRs may be involved in the process of CD8^+^ T cell differentiation in PD. Altogether, these results revealed a distinct cluster of terminal effector CD8^+^ T cells (C6 cluster), which exhibits obvious clonal expansion and cytotoxic differentiation by TCR activation in PD patients and is distinguished by expressing numerous genes involved in cell adhesion, migration, survival and cytotoxicity.

### A marked clonal expansion of cytotoxic CD4^+^ T cells in PD

**Fig. 4:**
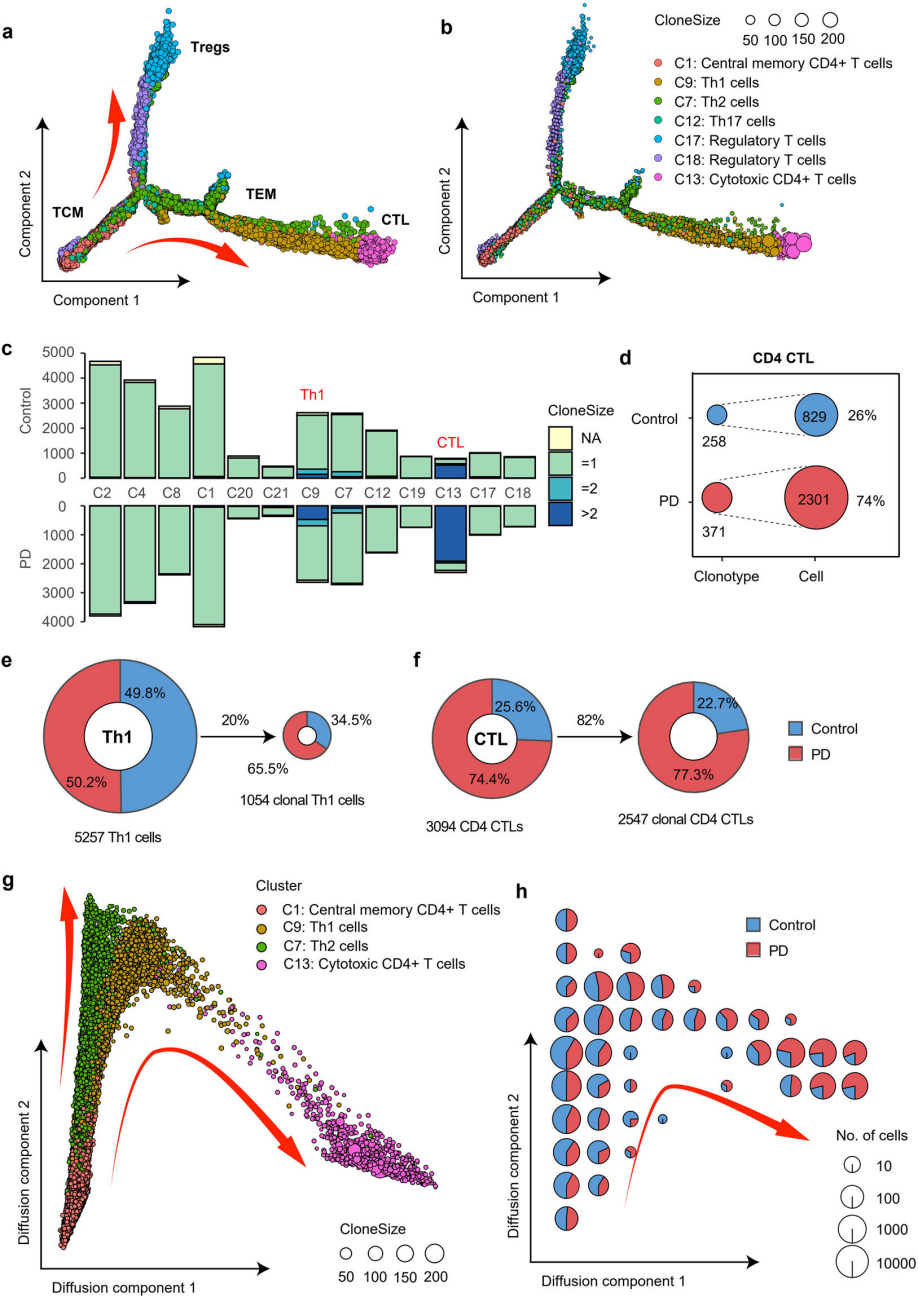
Clonal expansion of CD4^+^ T cells. **a** Pseudo ordering of CD4^+^ T cells by monocle. **b** Pseudo ordering of unique clonotypes in each cluster. Each dot represents a unique clonotype in each cluster. The size of the dot reflects the clone size in each cluster. The color of the dot distinguishes different clusters. The coordinates of each clonotype were calculated by the average coordinates of the cells in that clonotype. **c** Bar plots showing the distribution of clonally expanded CD4^+^ T cells in the blood of PD patients and healthy controls. Fisher’s exact test was used to identify clusters enriched with clonal expansion of T cells from PD patients. **d** Clonotype diversity of CD4 CTLs in PD patients and healthy controls. Only samples with scTCR-seq data were counted. **e** Pie charts showing the sample composition of total cells and clonally expanded cells in Th1 cells (C9 cluster), respectively. Only samples with scTCR-seq were counted. **f** Similar to **e**, pie charts showing the sample composition of total cells and clonally expanded cells in CD4 CTLs (C13 cluster), respectively. **g** Pseudo ordering of T_CM_, Th1 cells, Th2 cells and CD4 CTLs in diffusion trajectories using diffusion maps. **h** Cell sample composition corresponding to the diffusion trajectory in **g**. The diffusion trajectory was divided into 10 × 10 square grids according to the horizontal and vertical coordinates, and each pie chart reflects the sample composition of cells in each grid area.

CD4^+^ T cells are a large population of cells that play an important role in peripheral immunity in PD^11^. We annotated 8 major CD4^+^ T cell subtypes, including naïve CD4^+^ T cells (C2, C4 and C8 clusters), central memory CD4^+^ T cells (C1 cluster), cytotoxic CD4^+^ T cells (CD4 CTL, C13 cluster), Th1 cells (C9 cluster), Th2 cells (C7 cluster), Th17 cells (C12 cluster), Tfh cells (C19 cluster), and regulatory T cells (C17 and C18 clusters). Some highly expressed genes in each cluster were shown in Supplementary Fig. S4a. CD4 CTLs (C13 cluster) exhibited significantly higher expression of CD4 and several cytotoxic genes, such as GZMA, GZMB, GZMH and NKG7 (Supplementary Fig. S4a). There is no significant difference in the composition of CD4^+^ T cell subtypes between PD patients and healthy controls (Supplementary Fig. S4b–c). To understand the relationship among these CD4^+^ T cells, we constructed single-cell trajectories using R package Monocle 2 (version 2.14.0) (Fig. 4a). Central memory T cells (C1 cluster, T_CM_) were selected as the starting cell type of the differentiation (Fig. 4a). Consistent with the clustering analyses, we observed a process of transformation from central memory T cells (C1 cluster, T_CM_) to effector T cells (C9, C7 and C12 clusters, T_EM_) followed by CD4 CTLs (C13 cluster, CTL) (Fig. 4a). Regulatory CD4^+^ T cells (C17 and C18 clusters, Tregs) were reasonably located in a different branch (Fig. 4a). Larger clonotypes tend to be located at the end of the effector branch (Fig. 4b).

To gain insight into the clonal relationship among CD4^+^ T cells, we used Fisher’s exact test to identify PD-specific clonally expanded CD4^+^ T cell clusters. Compared to healthy controls, clonally expanded CD4^+^ T cells were significantly increased in Th1 cells and CD4 CTLs (C9 and C13 cluster) in the blood of PD patients (Fisher’s exact test, FDR = 8.58e−28 and 3.92e−14, respectively, Fig. 4c). Specifically, Th1 cells in the blood of PD patients accounted for 50.2% of total Th1 cells (C9 cluster), and this proportion increased to 65.5% when the background was reduced to clonally expanded Th1 cells (Fig. 4e). Regarding CD4 CTLs (C13 cluster), 74.4% of this population were from the blood of PD patients, and this percentage increased to 77.3% when the background was reduced to clonally expanded CD4 CTLs (Fig. 4f). CD4 CTLs tend to have larger clonotypes in PD patients with 371 clonotypes detected from 2301 cells (6.2 cells per clonotype), while the average clone size was 3.2 (258 clonotypes from 829 cells) in healthy controls (Fig. 4d). We used diffusion maps to further visualize the relationships among T_CM_, Th1, Th2 and CD4 CTLs (Fig. 4g). Both Th1 and Th2 cells originated from T_CM_ cells and began to differentiate in parallel. Thereafter, the differentiation trajectory separated, and some Th1 cells eventually transformed to CD4 CTLs (Fig. 4g). Larger clonotypes tend to distribute at the end of the CTL branch (Fig. 4g). The proportion of PD cells gradually increased along the trajectory (Fig. 4h). The average expression of 4 major cytotoxic genes GZMA, GZMB, PRF1 and NKG7, which are known to be abundant in CD4 CTLs^34,35^, increased along the differentiation trajectory of CD4 CTLs (Supplementary Fig. S5a, b). The evidence of TCR sharing further supported the state transition from Th1 cells to CD4 CTLs. In total, 81 clonotypes were identified with both Th1 cells and CD4 CTLs, such as clonotype28 and clonotype65 (Supplementary Fig. S5c). These results reveal that a group of CD4 CTLs derived from TCR-activated Th1 cells were significantly clonally expanded in PD patients.

Th1 cells could have cytotoxic effects on dopaminergic neurons by releasing IFNγ, which activates and recruits other immune cells to amplify local inflammation^6^. It has also been reported that CD4^+^ T cell mediated dopaminergic toxicity does not require the expression of IFNγ in a mouse model of PD^11^, suggesting the presence of cytotoxic CD4^+^ T cells infiltration in the central nervous system. Our study reveals that both Th1 and CD4 CTL were significantly clonally expanded by TCR-dependent activation in the blood of PD patients, suggesting that these two cell types in the blood may be the source of central infiltrating CD4^+^ T cells^36^. Inhibitors that direct or indirect target of these T cell types may block the immune response in PD patients by preventing T cell proliferation^6^.

### Antigen-specific T cells and candidate antigenic epitopes in PD

Increasing evidence indicates that abnormal processing of self-proteins can produce antigens in PD^37^. T cells recognize these antigens, coordinate local innate immune responses, and drive dopaminergic neuronal death by activating immune pathways^5^. α-Synuclein (α-syn) is a presynaptic neuron protein that is genetically and pathologically related to PD^38^. Recent studies have shown that fibrils of α-syn can recruit peripheral immune cells prior to neurodegeneration in the rat brain^39^. Misfolded α-syn is not only prevalent in the central nervous system but can also cause peripheral immune responses^10^. A group of peptides derived from α-syn have been reported as epitopes driving T cell responses in PD patients^8^. In addition, the mitochondrial antigen presentation pathway is also associated with adaptive immunity in PD^40^. Recognition of antigen-specific T cells is crucial for understanding the adaptive immune response in PD.

**Fig. 5:**
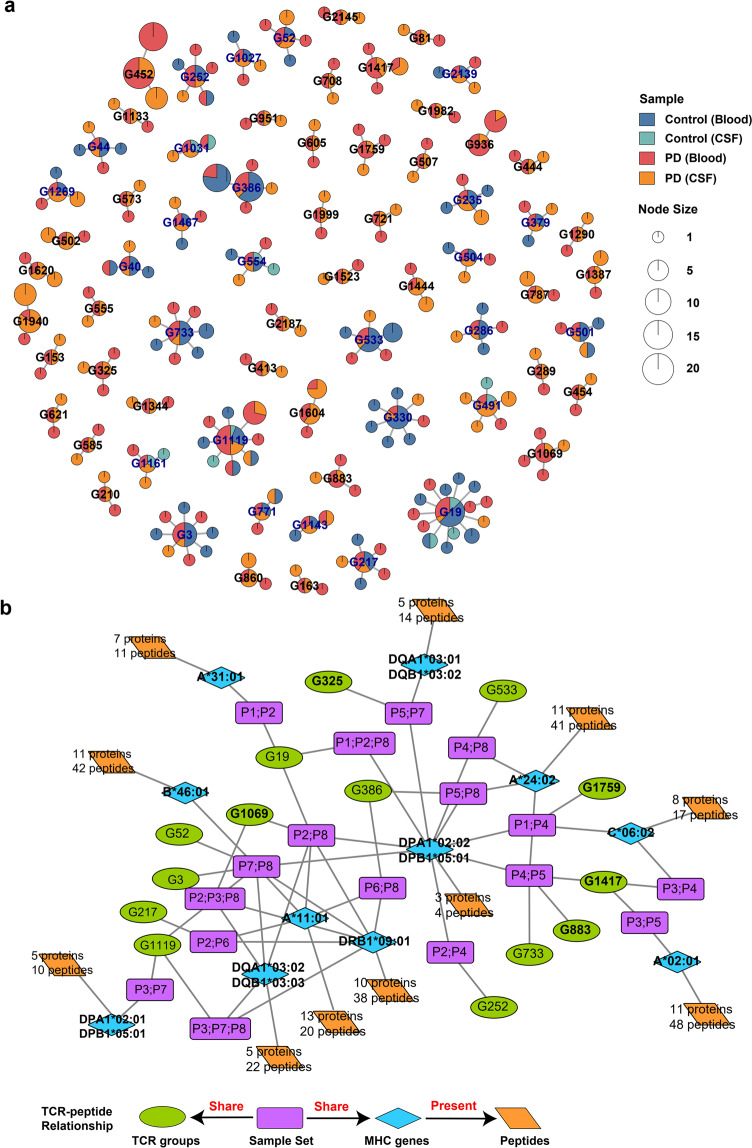
Antigen-specific TCR groups in PD. **a** Network plot showing the 67 TCR specificity groups with at least one cell from the blood and one cell from CSF of PD patients. Most of the TCR groups were found only in PD patients. **b** Strong sample sharing relationships between 14 TCR specificity groups and 11 HLA alleles. For example, G1759 contains TCRs from patient P1 and P4, and patient P1 and P4 also have the same allele of HLA-C06:02. Thus, the relationship between G1759 and HLA-C06:02 can be constructed through the overlap of sample sets (such as P1 and P4).

TCR clustering based on CDR3 sequence similarity is an effective approach to identify antigen-specific T cells^41,42^ as TCRs sharing similar motifs from distinct individuals may also share antigen specificity. In total, we obtained 110,912 βCDR3s from 113,690 T cells and performed pairwise alignment. We used an ultrafast algorithm, iSMART^43^, specifically designed to handle large amount of TCR clustering and detected 1778 TCR specificity groups (Supplementary Table S7). To identify PD-specific TCRs, we screened 67 TCR specificity groups with at least one TCR from blood and one TCR from CSF of the PD patients (Fig. 5a, Supplementary Table S7). These groups were considered as candidates for PD-specific TCRs, most of which were found exclusively in PD patients (Fig. 5a).

The identification of PD-specific TCRs also enables us to further uncover the candidate antigenic epitopes from the PD-related proteins, such as α-syn. We used several steps to find the relationship between PD-specific TCRs and potential antigenic epitopes. First, high resolution HLA typing was obtained from the whole genome sequencing data of our 8 PD patients (Supplementary Table S6). Second, we searched NCBI protein database with keywords of ‘alpha-synuclein’ and ‘mitochondrial’ and obtained all the α-syn and mitochondrial protein sequences. After removing the redundancy, these protein sequences were separated into 9-mer and 15-mer peptides to predict their binding affinity with MHC I and MHC II alleles, respectively. Finally, we used samples shared by MHC genes and TCRs to construct the relationship between MHC-peptides and TCRs (Fig. 5b). A relatively strong sample sharing relationship was noted between 14 TCR specificity groups and 11 HLA alleles (Fig. 5b). These HLA alleles were predicted to bind to at least one peptide from α-syn or mitochondrial proteins (Fig. 5b; Supplementary Table S7). Fortunately, two of our predicted peptides ‘KTKEGVLYVGSKTKE’ and ‘GKTKEGVLYVGSKTK’ have been reported to drive helper and cytotoxic T cell responses in PD patients^8^ (Supplementary Fig. S6). In summary, we used TCR clustering and machine learning to screen a group of PD-specific TCRs and their candidate epitopes, providing potential targets for blood and cerebrospinal fluid T cells to participate in neuronal degeneration.

### Possible mechanism of cytotoxic T cells passing through the BBB in PD

The blood-brain barrier (BBB) is a physical barrier formed by endothelial cells to prevent blood proteins, antibodies and immune cells from penetrating into the brain parenchyma^44^. However, under the continuous action of chronic inflammation, the tight junctions between endothelial cells are weakened or destroyed, thus allowing antibodies or immune cells to pass through^45^. Postmortem studies of the brain have confirmed that the infiltration of lymphocytes into the brain contributes to the neurodegeneration of PD^9,11,46^. Numerous adhesion molecules are involved in the recruitment of leukocytes, especially lymphocytes, into the central nervous system (CNS) during inflammation. The integrin leukocyte function-associated antigen-1 (LFA-1) plays a key role in leukocyte adhesion cascade by binding ICAM-1 (and ICAM-2) on the surface of endothelial cells^47^. Very late activation antigen-4 (VLA-4) mediates the adhesion of lymphocytes and monocytes to VCAM-1 on the surface of activated endothelial cells^48^. Macrophage-1 antigen (MAC-1) binding to ICAM-1 (and ICAM-2) regulates intravascular crawling^49^. In addition, several chemokines and their receptors are associated with the recirculation of effector T cells to the BBB. Chemokine receptors (such as CXCR4) on rolling leukocytes interact with chemokines (such as CXCL12) on endothelial cells, activating several signaling pathways (such as PI3K, PLC, RAS- and RHO-family GTPase, and MAPK) and promoting an opened integrin conformation^50–52^. Selectins (SELE, SELP) and their counter ligands (SELPLG) dependent rolling is the earliest observable event of leukocyte recruitment to inflammatory tissues^53^, which plays a critical role in the recruitment of CD8^+^ cells in brain vessels of patients with multiple sclerosis during acute attacks^54^.

**Fig. 6:**
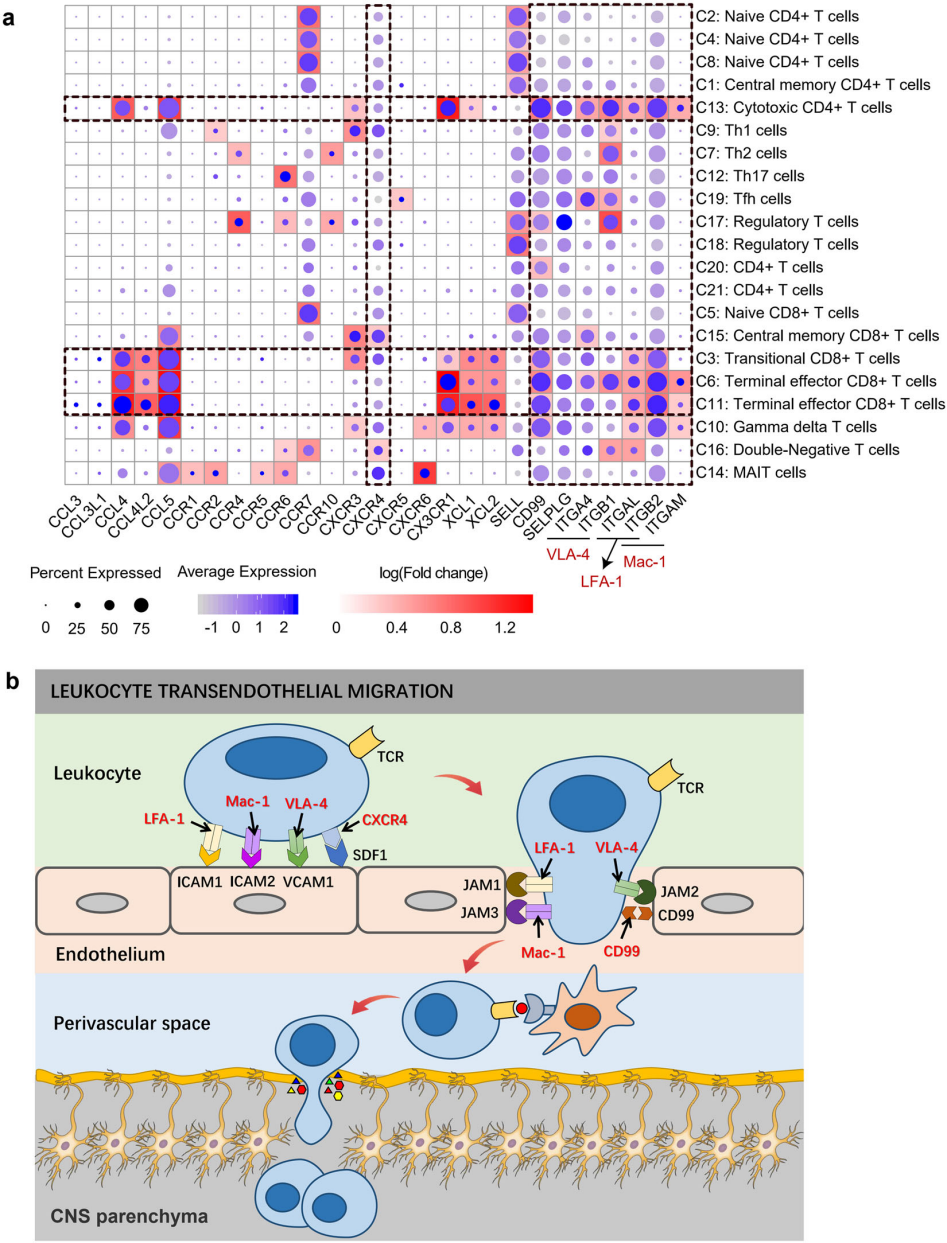
Cell migration and adhesion-related genes may be involved in lymphocytes penetrating the BBB in the PD. **a** A global view of the expression profiling of genes related to cell migration and adhesion in each cluster. The size of the dot corresponds to the percentage of cells expressing the gene in each cluster, and the color represents the average log normalized gene expression. Background heatmap shows the fold change in gene expression between each cluster and other clusters. Fold change was calculated using the function FindAllMarkers in R package Seurat with default parameters. Only positive markers (logFC > 0) in each cluster are shown in the heatmap. **b** Possible mechanism of cytotoxic T cells passing through the BBB in PD. Depiction of lymphocyte migration across endothelial cells was based on the leukocyte transendothelial migration pathway (KEGG pathway: hsa04670). During inflammation, adhesion molecules, chemokines and their receptors mediate the arrest, polarization, directed crawling and endothelial passage of lymphocytes on endothelial cells. Once T cells cross the BBB endothelium, T cells may recognize their cognate antigens and become reactivated behind the BBB. The amplified inflammatory response leads to parenchymal basement membrane damage. Finally, activated T cells enter the brain parenchyma to participate in central nervous system damage.

We assessed numerous molecules related to cell migration and adhesion and found that many molecules related to BBB penetration were highly expressed in cytotoxic T cells (Fig. 6a; Supplementary Table S3). Integrin family genes (VLA-4, LFA-1, Mac-1) exhibited relatively high expression in transitional CD8^+^ T cells (C3 cluster), terminal effector CD8^+^ T cells (C6, C11 clusters) and CD4 CTLs (C13 cluster) (Fig. 6a; Supplementary Table S3). Other cellular chemokines, adhesion molecules and their receptors, such as CCL4, CCL5, CX3CR1, CD99 and SELPLG, were also widely and relatively highly expressed in these cytotoxic T cells (Fig. 6a). Some genes also showed significantly upregulated expression in PD patients (Supplementary Table S4). These genes were significantly enriched in the leukocyte transendothelial migration pathway (KEGG: hsa04670), which may represent a possible mechanism by which cytotoxic T cells pass through the BBB in PD^55^ (Fig. 6b).

## Discussion

Numerous postmortem studies have confirmed the presence of lymphocyte infiltration in the brain of PD patients^11,46^. High levels of activated T cells have also been detected in the cerebrospinal fluid of PD patients^56^. Moreover, lymphocyte infiltration is not a random event caused by damage to the BBB but targeted migration to the vicinity of dopaminergic neurons in the brain of PD patients^9,11^. Given the chronic inflammatory nature of PD, T cell immunity may be important for disease onset. Therapies targeting T cells can reduce neurodegeneration and motor behavior disorders in animal models of PD^57^. The study of T cell populations in peripheral blood and cerebrospinal fluid of PD patients will further improve our understanding of the immune pathogenesis of PD.

In this study, we conducted integrative computational analyses to investigate the immunological changes in the blood and cerebrospinal fluid of PD patients compared to healthy controls. We identified a distinct cluster of terminal effector CD8^+^ T cells significantly clonally expanded in PD patients, which derived from central memory CD8^+^ T cells by TCR-dependent activation and upregulated both cell adhesion (ITGAM, ITGB1, etc.) and cell survival (PRSS23, SPON2, ZNF683) markers. Notably, we reported a group of cytotoxic CD4^+^ T cells (CD4 CTLs) significantly clonally expanded in PD patients, which may be a source of central infiltrating cytotoxic CD4^+^ T cells. Evidence of TCR sharing further supports their differentiation from Th1 cells. These cytotoxic CD8^+^ and CD4^+^ T cell populations are strong candidate for potential involvement in the pathogenesis of PD. In addition, we grouped TCRs by CDR3 sequence similarity and provided potential TCR-antigen relationships by MHC-peptide prediction and overlap analyses between samples with the same MHC alleles and TCR groups. Two of our predicted peptides ‘KTKEGVLYVGSKTKE’ and ‘GKTKEGVLYVGSKTK’ have been reported to drive helper and cytotoxic T cell responses in PD patients^8^ (Supplementary Fig. S6). These findings provide evidence of convergent selection in PD. Future efforts can be made to assess the antigenicity of the predicted epitopes using effector T cells transfected with synthetic TCRs, by testing their cytokine secretion with immunospot assay upon antigen stimulation.

It is estimated that approximately 4 × 10^11^ T cells circulate in the adult human body^58^. Cells detected by single-cell sequencing are only the tip of the iceberg and do not completely represent all the immune diversity. It is difficult to find common TCRs from different individuals. It is a good idea to use the similarity of βCDR3 to identify common antigen-specific TCRs in different individuals, but large-scale TCR repertoire sequencing data are still needed to obtain more accurate results. In addition, the diversity of MHC alleles in the population also hinders the identification of antigen-specific T cells shared by the population. Moreover, the limited number of cells detected in cerebrospinal fluid data used in this study also hinders the identification of common clonal T cells between blood and cerebrospinal fluid. In the future, large-scale single cell sequencing data of lymphocytes in blood and cerebrospinal fluid are still necessary, and mixed TCR immune repertoire sequencing data are also needed to assess the diversity of lymphocytes as much as possible.

## Materials and methods

### Human research participants

Eight PD patients (P1–P8) aged 50–70 years with stable and effective L-dopamine medication were recruited in this study. None of the candidates had significant somatic disorders, such as tumor, autoimmune disorders and chronic diseases, as well as psychiatric co-morbidities, including mild cognitive impairment (MCI) and dementia. Six age-matched healthy controls (N1–N6) were also recruited. All participants were procured from the First Affiliated Hospital of Harbin Medical University. This study was approved by the Ethics Committee in the First Affiliated Hospital of Harbin Medical University (Approval number: No. 201985). Informed consent was obtained from all participants.

### Publicly available datasets

In this study, an additional seven healthy controls (N7–N13) were included to enrich the datasets of health controls. Specifically, N7 and N8 were downloaded from the official website of 10× genomics with both scRNA-seq and scTCR-seq data (https://support.10xgenomics.com/single-cellvdj/datasets), and N9–N13 (aged in their 50 to 80 years) were downloaded from Hashimoto et al.^35^ with only scRNA-seq data.

In addition, publicly available single-cell immune profiling datasets from cerebrospinal fluid^16^, including 6 PD patients (PD1–PD6) and 9 healthy controls (HC1–HC9), were downloaded and used to better understand clonal expansion of lymphocyte T cells in PD. The average age of CSF samples was 68.71 (8.61 SD). All of these published single-cell transcriptome and immune sequencing data were generated on the 10× Genomics platform.

### Blood sample collection and preparation

Fresh blood samples from eight PD patients (P1–P8) and six age-matched healthy controls (N1–N6) were collected and followed by density gradient centrifugation on Percoll to isolate human peripheral blood mononuclear cells (PBMCs). CD3^+^ T cells were then isolated from PBMCs by fluorescence-activated cell sorting (FACS) analysis.

### Bulk DNA isolation and sequencing

Genomic DNA of blood was extracted using Invitrogen Genomic DNA Extraction Kits according to the manufacturer’s specification. The concentrations of DNA were quantified using a NanoDrop instrument (Thermo) and the qualities of DNA were evaluated with agarose gel electrophoresis. DNA libraries were constructed by fragmenting genomic DNA (approximately 0.1–1 µg) using the NEBNext Ultra DNA Library Prep Kit. Finally, DNA libraries were sequenced on the Illumina Novaseq 6000 with 150-bp paired end (PE150).

### Single-cell 5′ and V(D)J sequencing

Single-cell 5′ and V(D)J libraries were prepared following the protocol provided by the 10× genomics Chromium Single Cell Immune Profiling Solution. Briefly, CD3^+^ T cell suspensions (400–1000 living cells per microliter determined by CounterStar) were loaded on a Chromium Single Cell Controller (10× Genomics) to generate single-cell gel beads in emulsion (GEMs) using Chromium Single Cell V(D)J Reagent Kits. Captured cells were lysed, and the released RNAs were barcoded through reverse transcription in individual GEMs. Each single-cell 5’ and V(D)J libraries were sequenced by the Illumina Novaseq 6000 using 150 paired-end reads.

### HLA genotyping

High accuracy of human leukocyte antigen (HLA) allotype (i.e., a set of HLA alleles of an individual) of eight PD patients were characterized by HLA-HD^59^ based on the information from whole genome sequencing. First, we created an HLA allele dictionary from the current allele information to increase the completeness of applicable alleles. Then, high-quality reads were mapped to the HLA allele dictionary using bowtie2^60^. Finally, suitable pairs of HLA alleles were selected by calculating a score based on weighted read counts^59^.

### Preprocessing of single-cell transcriptome data

Single-cell transcriptome data were preprocessed using the following steps: First, we used UMI-tools^61^ to identify cell barcodes and UMIs. Then, cell barcodes and UMIs were appended to the read names to distinguish different cells and different RNA molecules. Read adapters were trimmed using cutadapt^62^. High-quality reads were then mapped to the GRCh38 (Release-92) human reference genome using STAR^63^. The number of reads mapping to each genomic gene were counted using featureCounts^64^. Samtools^65^ were used to sort and index BAM files, which stores mapped reads in a standard and efficient manner. Then, the UMI-corrected molecular counts were calculated using UMI-tools^61^. Finally, a local Perl script was used to construct a combined gene expression matrix containing all the sequenced samples.

### Cell quality control

Real cells from empty droplets were called using the emptyDrops function from R package dropletUtils, which assesses whether the RNA content associated with a cell barcode is significantly distinct from the ambient background RNA present within each sample^66,67^. Cells with FDR ≤ 0.01 (Benjamini-Hochberg corrected) were considered for further analysis. Then, low-quality cells were identified and removed using the isOutlier function in R package scater^68^, which identifies outliers based on the median absolute deviation (MAD)^69^. Cells were claimed as low-quality cells if: (1) The cell library size (total UMI counts) is smaller than 3 MADs; (2) The number of detected genes is smaller than 3 MADs; (3) The proportion of mitochondrial gene counts is bigger than 3 MADs. Please see Zhang et al.^70^ for details. Doublets were identified and filtered by DoubletFinder^71^ with the expected doublet rate of 0.075. Finally, genes with more than 1 transcript in at least two cells were retained for further analysis.

### Dataset integration and unsupervised clustering

Batch effects were removed, and datasets from each sample were integrated using the standard Seurat v3 integration workflow^18,19^. First, raw counts of each sample were normalized using a global-scaling normalization method NormalizeData in R package Seurat^18,19^. This method normalizes the gene expression values for each cell by the total UMI counts in the sample, then multiplies this value by a scale factor (10,000 by default), and log-transforms the result. Highly variable genes were identified in each sample using FindVariableFeatures function in Seurat^18,19^. To identify shared cell states that are present across blood and cerebrospinal fluid samples, ‘anchors’ between pairs of datasets were identified and used to harmonize the datasets. Finally, the cell-cycle score was calculated using CellCycleScoring function and regressed during data scaling using the ScaleData function in Seurat^18,19^.

We used a graph-based clustering approach implemented in Seurat^18,19^ to perform unsupervised clustering of all T cells. First, principal component analysis was computed based on the scaled expression of variable genes. Then, 15 principal components were used to construct a KNN graph using the FindNeighbors function in Seurat^18,19^, in which the edge weights between any two cells were based on the shared overlap in their local neighborhoods (Jaccard similarity). Finally, cells were clustered using the FindClusters function in Seurat^18,19^, which used the Louvain algorithm to iteratively group cells together with the goal of optimizing the standard modularity function. Additional K-means clustering was further used to classify cytotoxic T cells into CD8 CTLs and CD4 CTLs (C6 and C13 clusters). Cluster with less than 500 cells were removed from downstream analysis.

Based on the gene expression profiling, a dimensionality reduction method called Uniform Manifold Approximation and Projection (UMAP) was used to visualize T cells in a two-dimensional space. UMAP projections were generated by RunUMAP function in Seurat^18,19^ based on the first 15 principal components.

### Cell type annotation

Cluster biomarkers were identified using the FindAllMarkers procedure in Seurat^18,19^, which identified differentially expressed genes for each cluster using a Wilcoxon Rank Sum test. The R package SingleR^72^ was then used to further annotate single cells by leveraging reference transcriptomic datasets of pure cell types to infer the cell of origin of each single cell independently. Three bulk RNA-seq datasets of purified immune cells (The Database for Immune Cell Expression (Schmiedel et al.^20^), Monaco Immune Cell Data (Monaco et al.^21^), the Human Primary Cell Atlas (Mabbott et al.^22^), BLUEPRINT database (Martens et al.^23^) and Novershtern Hematopoietic Data (Novershtern et al.^24^) were selected as reference datasets for single-cell annotation.

Cell clusters were manually annotated by checking the expression of classic marker genes and single-cell annotation by the purified bulk RNA-seq datasets. For example, C2, C4, C8 were annotated as Naïve CD4^+^ T cells based on two evidence: (1) C2, C4, C8 highly expressed naïve T cell markers SELL, CCR7, TCF7 and LEF1 (Fig. 1c); (2) More than 90% of cells from C2, C4, C8 were annotated as Naïve CD4^+^ T cells by bulk dataset Martens et al.^23^ (Supplementary Fig. S1a).

### Differential expression analysis

Differential expression analysis was conducted by using the FindMarkers function in Seurat^18,19^ with default parameters, which used normalized gene expression values as input. To calculate the logFC value, the average expression values in each group added by 1 (where 1 represents a pseudocount) were divided between two groups and then log-transformed. Genes were claimed as differentially expressed if: (1) Genes should be detected in at least 10% of the cells in either of the two groups; (2) The threshold of logFC is the default value of 0.25; (3) Bonferroni adjusted *P*-value is less than 0.05. Differentially expressed genes (DEGs) between the blood of PD patients and healthy controls as well as cluster biomarkers of each cell cluster were combined to evaluate the role of cell clusters in the immune response of PD.

### Single-cell trajectory analysis

Monocle 2 (version 2.14.0) was used to investigate transcriptional and functional trajectories of CD4^+^ T cell clusters (Fig. 4a). Only 7 CD4^+^ T cell clusters were selected to construct the trajectory due to the limitation of the number of cells processed by Monocle. Given that the direction of pseudotime is arbitrary, we selected central memory CD4^+^ T cells as the beginning of the trajectory.

Diffusion maps represent a more advanced trajectory inference method, which was introduced by Ronald Coifman and Stephane Lafon^73^, and the underlying idea is to assume that the data are samples from a diffusion process. Diffusion maps are efficient, scalable and robust and provide better details of cell trajectory^74,75^. We choose diffusion maps implemented by R package destiny^75^ to analyze the trajectory of some specific clusters, such as CD8 CTLs (Fig. 3c) and CD4 CTLs (Fig. 4g). Central memory T cells were used to determine the beginning of the trajectory.

### Single-cell V(D)J data processing

Single-cell V(D)J data was processed using Cell Ranger (10× Genomics, version 3.1.0) with –reference = refdata-cellranger-vdj-GRCh38-alts-ensembl-3.1.0 for each sample. Paired α and β CDR3 sequences from blood and cerebrospinal fluid were pooled together to identify common clonotypes across samples. Cells with the same CDR3 sequence for both the α-chain and the β-chain were considered the same clonotype.

### Antigen-specific TCR groups analysis

Clustering of TCRs based on CDR3 similarity is an effective approach to identify antigen-specific T cells^41,42^ given that TCRs sharing similar motifs from distinct individuals may also share antigen specificity. We grouped all the βCDR3 sequences from blood and cerebrospinal fluid samples and identified antigen-specific TCR groups using iSMART^43^, which performs a specially parameterized pairwise local alignment on T cell receptor CDR3 sequences to group them into antigen-specific clusters. For a given group with high similarity, the antigen-specific TCR group needs to meet the following conditions: (1) Only one amino acid mismatch is allowed on CDR3; (2) Only one insertion or deletion is allowed on CDR3; (3) V genes within the group should be the same.

### HLA antigen presentation prediction

Prediction of HLA antigen presentation is a key step in identifying antigen epitopes and understanding adaptive immunity of PD. The accumulation of abnormal forms of α-syn is a trigger of PD. Recent evidence suggests a strong relationship between α-syn and adaptive immune system, which may lead to downstream neurodegeneration^76^. Mitochondrial damage that causes mitochondrial proteins to be presented on the neuron surface also leads to the activation of adaptive immune responses in PD^40^. Therefore, we focused on these two types of proteins to screen the potential epitopes that can be presented by patients’ MHC alleles. To achieve this, we first searched the NCBI protein database with the keywords of ‘alpha-synuclein’ and ‘mitochondrial’ and obtained all the α-syn and mitochondrial protein sequences. After removing the redundancy, these protein sequences were separated into 9-mer and 15-mer peptides using sliding windows to predict their binding affinity with MHC I and MHC II alleles using NetMHCstabpan^77^ and NetMHCIIpan^78^, respectively.

### Measures of TCR diversity

TCR diversity was calculated based on the D50 value^79^, which is the percentage of dominant T cell clonotypes that account for the cumulative 50% of the total CDR3s counted in the sample^79^. The more diverse the TCR repertoire, the closer the value is to 50.

The D50 value is defined as follows:

$$D50^j = \frac{{argmin_k\left( {\mathop {\sum }\nolimits_{i = 1}^k N_i^j - \frac{1}{2}\mathop {\sum }\nolimits_{i = 1}^n N_i^j} \right) \times 100}}{n}$$

where n is the total number of unique CDR3s, and $N_i^j$ is the frequency of the i-th CDR3 in sample j in the following order:

$$N_1^j \ge N_2^j \ge \ldots N_i^j \ge N_{i + 1}^j \ge \ldots \ge N_n^j$$

## Supplementary information

Supplementary Information

Supplementary Table S1. Summary of single-cell RNA and TCR sequencing data.

Supplementary Table S2. Statistics on some features of MAIT and gdT cells in each cluster.

Supplementary Table S3. Marker genes for each cluster.

Supplementary Table S4. Differentially expressed genes for each cluster between the blood of Parkinson’s patients and healthy controls.

Supplementary Table S5. Summary of single-cell TCR sequencing.

Supplementary Table S6. HLA genotyping and MHC-peptide binding prediction.

Supplementary Table S7. Antigen specificity TCR clustering and potential Parkinson-specific TCR groups.

## Data Availability

All single-cell RNA sequencing data have been deposited in the zenodo under accession URL https://zenodo.org/record/3993994.
