# Supplementary material for: Single-cell transcriptome and TCR profiling reveal activated and expanded T cell populations in Parkinson’s disease
Source: Cell Discov. 2021 Jul 20;7:52. doi: 10.1038/s41421-021-00280-3 (PMC8289849; doi:10.1038/s41421-021-00280-3)
Supplement: Supplementary file 1 — Supplementary Information [file 41421_2021_280_MOESM1_ESM.pdf]

Supplementary Figures

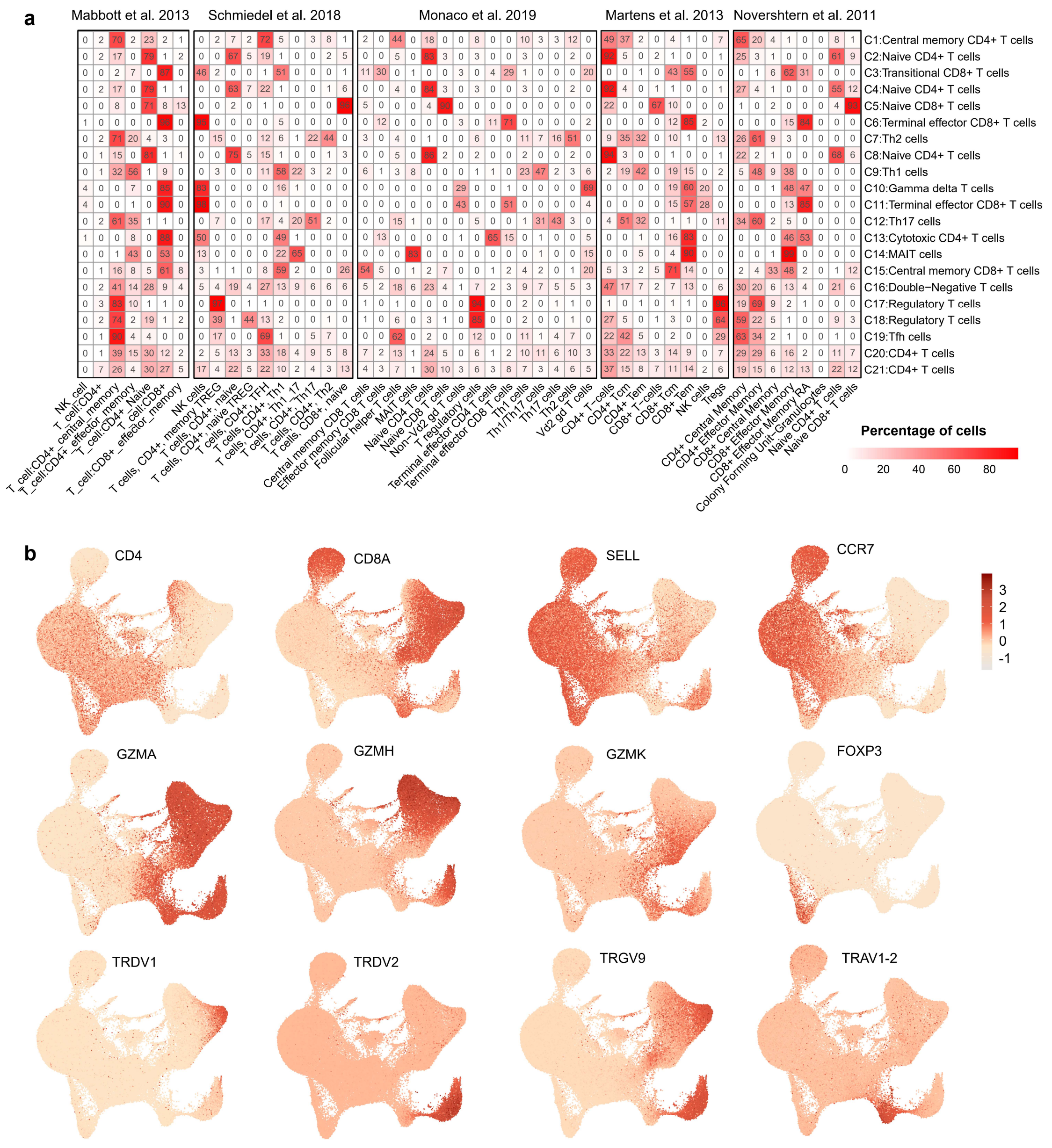

Supplementary Fig. S1 Cell type annotation by classic marker genes and purified bulk RNA-seq datasets. a Heatmap showing the percentage of cells in each cluster annotated by purified bulk RNA-seq datasets. Five bulk RNA-seq datasets of purified immune cells, The Database for Immune Cell Expression (Schmiedel et al. 2018[1]), Monaco Immune Cell Data (Monaco et al. 2019[2]), the Human Primary Cell Atlas (Mabbott et al. 2013[3]), BLUEPRINT database (Martens et al. 2013[4]) and Novershtern Hematopoietic Data (Novershtern et al. 2011[5]), were selected as reference datasets. Spearman correlation between single cell and bulk RNA-seq expression profiles were computed, and the cell labels were transferred from bulk RNA-seq datasets to every single cell. Each dataset was used separately to calculate the correlation. The labels on the heatmap is the percentage of cells annotated by the corresponding dataset. In most cases, a cluster was annotated by the labels of majority of cells from that cluster. **b** UMAP projects of several markers to help distinguish different types of T cells.

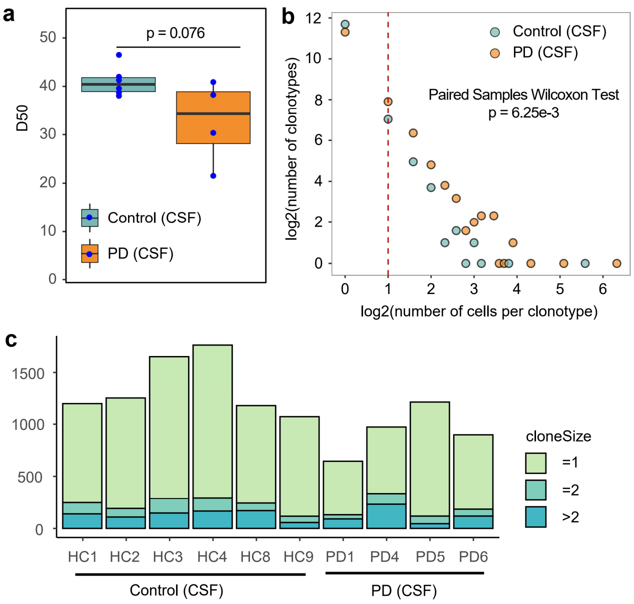

Supplementary Fig. S2 Single-cell immune profiling of T cells in the cerebrospinal fluid of PD patients and healthy controls. a TCR diversity comparison between samples in cerebrospinal fluid. TCR diversity was measured by D50, which is proven robust for the sequencing library size. **b** The association between the number of T cell clonotypes and the number of cells per clonotype. Downsampling was used to avoid bias caused by the total number of detected T cells between PD patients and healthy controls. The dashed line separates nonclonal and clonal cells, with the latter identified by repeated usage of αβTCRs. **c** Clonal composition of T cells in samples of cerebrospinal fluid based on clone size (= 1, = 2 and > 2). Notes: samples with less than 100 cells were not counted.

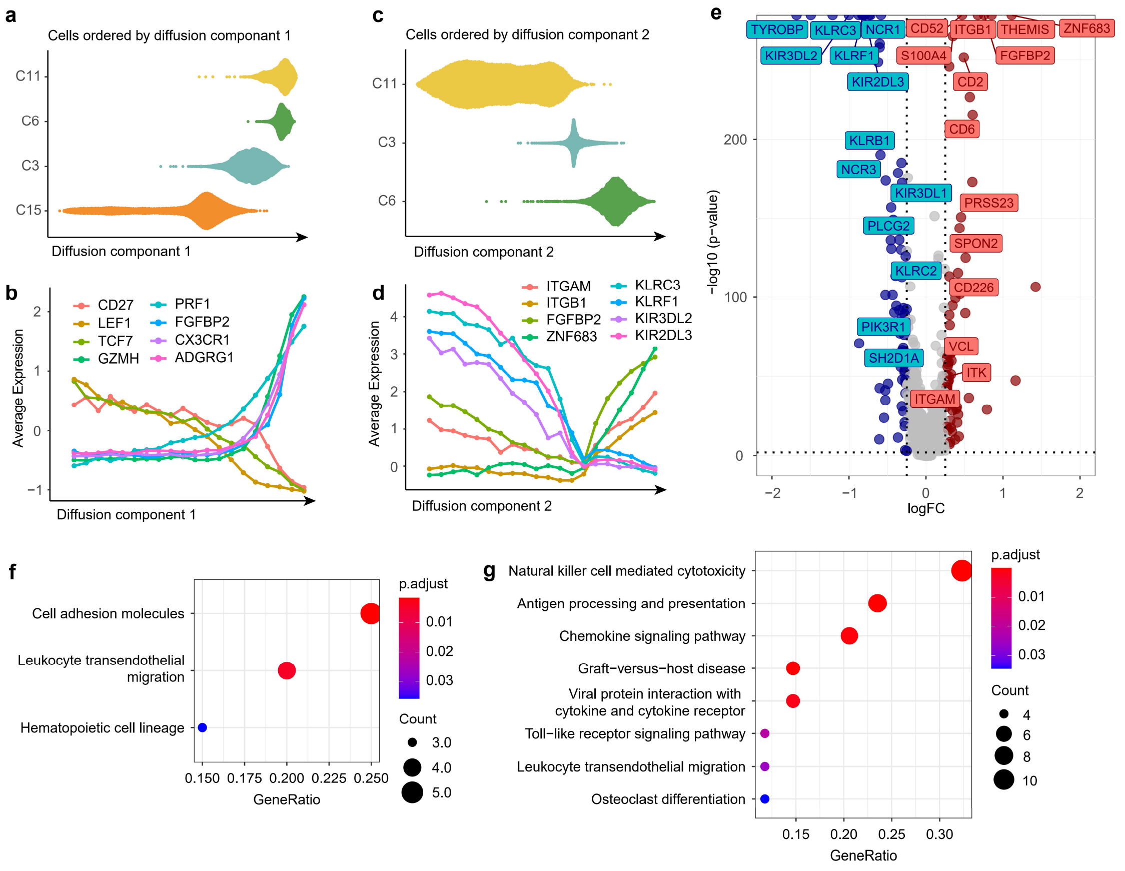

Supplementary Fig. S3 Cellular composition and gene expression characteristics of cytotoxic CD8$^+$ T cells. **a** Cells ordered by the first diffusion component. **b** Traceplot of T cell activation signatures for all CD8$^+$ T cells along the activation component (the first diffusion component). Cells were projected along the component (x-axis), and the dot indicates the moving average of gene expression using a sliding window of length equal to 5% of the x-coordinate range. **c** Similar to a, cells ordered by the second diffusion component. **d** Similar to b, traceplot of some differentially expressed genes between C6, C3 and C11 clusters for all effector CD8$^+$ T cells along differentiation component (the second diffusion component). **e** Volcano plot showing differentially expressed genes (DEGs) between two terminal effector CD8$^+$ T cell clusters C6 and C11. The x-axis represents the fold change between groups, and the y-axis represents *P* values. *P* values were estimated using two-sided Wilcoxon test, and FDR was corrected using BH. Genes upregulated in cluster C6 are noted in red, and genes upregulated in cluster C11 were marked in blue. **f** Dot plot showing the results of KEGG pathways enrichment analyses performed for C6 up-regulated genes. **g** Dot plot showing the results of KEGG pathways enrichment analyses performed for C11 up-regulated genes.

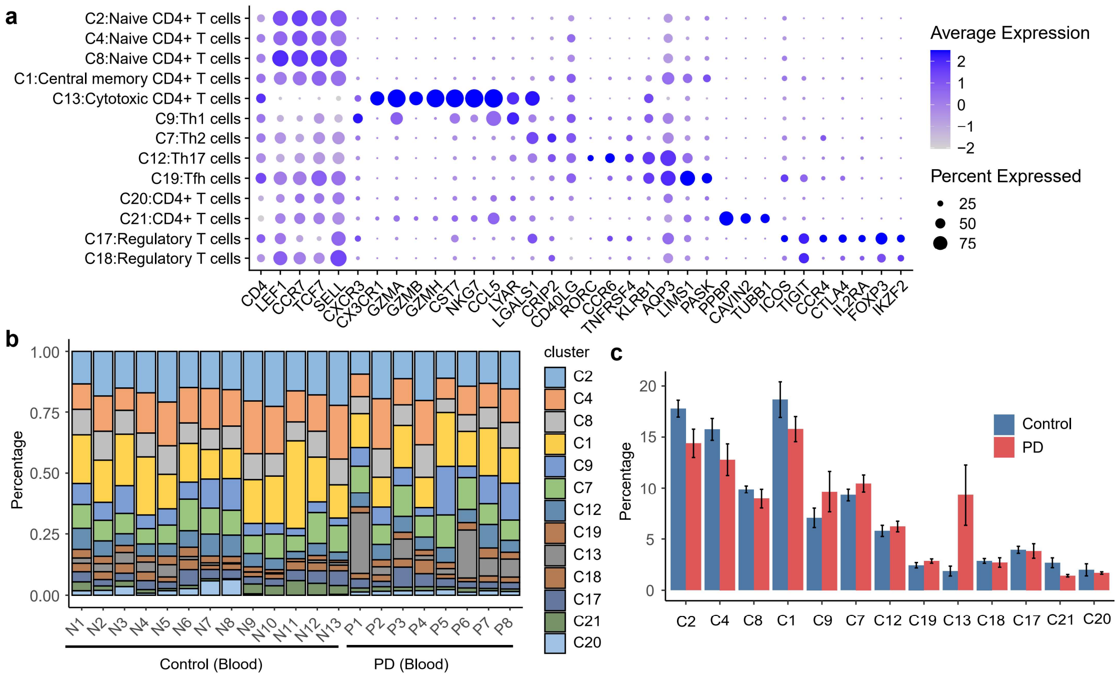

Supplementary Fig. S4 Gene expression pattern and cellular composition of CD4^{+} T cells. a Dot plot showing the expression of highly expressed genes in each CD4^{+} T cell cluster. The size of the dot corresponds to the percentage of cells expressing the gene in each cluster, and the color represents the average log normalized gene expression. **b** Percentage of CD4^{+} T cells in each sample. No significant difference in all CD4^{+} T cell subsets was observed between PD patients and healthy controls. **c** Bar plot showing the percentages for different CD4^{+} T cell subsets identified from single cell analysis. Error bar represents standard error of the mean.

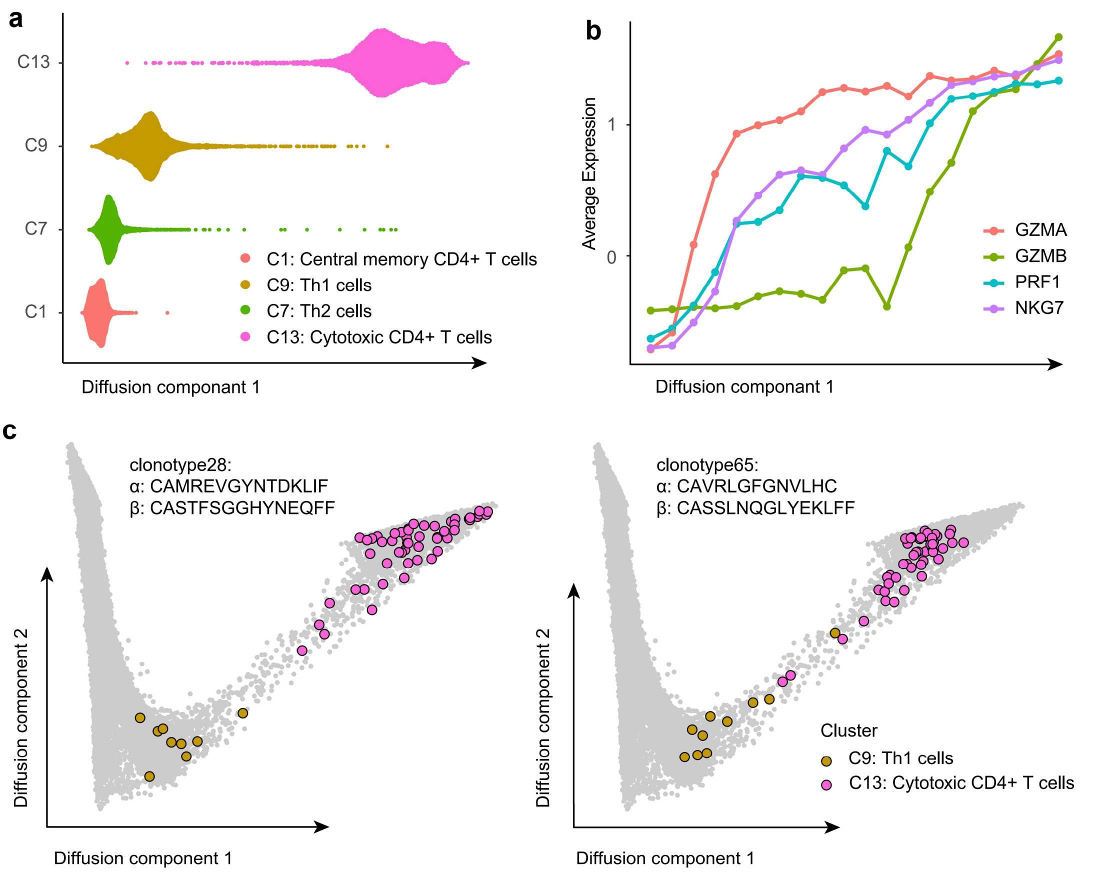

Supplementary Fig. S5 Pseudotime ordering and clonal expansion of CD4 CTLs in PD patients. **a** Cells ordered by the first diffusion component. CD4 CTLs are located at the end of differentiation. **b** Traceplot of cytotoxic genes GZMA, GZMB, PRF1 and NKG7 along the differentiation trajectory (the first diffusion component). Cells were projected along the component (x-axis), and the dot indicates the moving average of gene expression using a sliding window of length equal to 5% of the x-coordinate range. **c** Examples of clonal expansion of CD4^{+} T cells in different cell types. Cells from 2 clonotypes (clonotype28 and clonotype65) were highlighted in the diffusion trajectory. Each dot represents a cell colored based on its cluster.

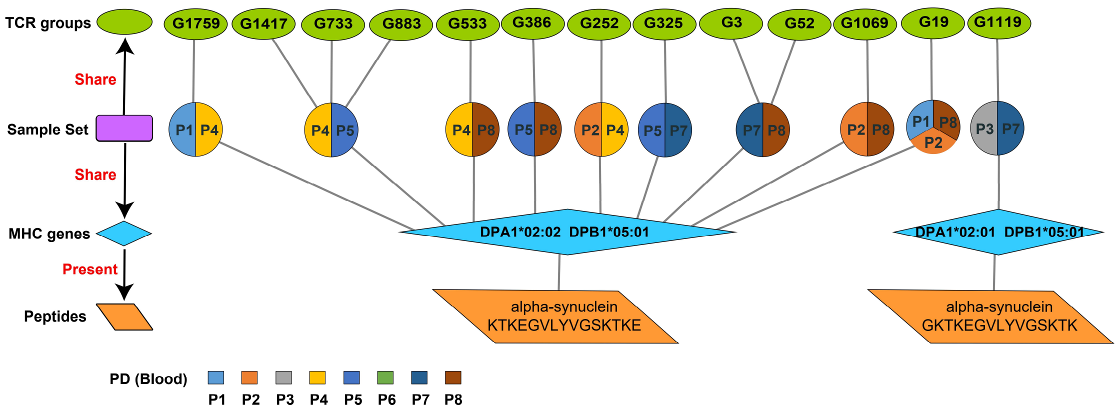

Supplementary Fig. S6 Network showing relationship between 13 TCR groups and 2 peptides presented by MHC II alleles "HLA-DPA1*02:02 HLA-DPB1*05:01" and "HLA-DPA1*02:01 HLA-DPB1*05:01" of patients with PD. The peptides KTKEGVLYVGSKTKE and GKTKEGVLYVGSKTK were confirmed in published articles.

Supplementary Tables

Supplementary Table S1. Summary of single-cell RNA and TCR sequencing data.

Supplementary Table S2. Statistics on some features of MAIT and gdT cells in each cluster.

Supplementary Table S3. Marker genes for each cluster.

Supplementary Table S4. Differentially expressed genes for each cluster between the blood of PD patients and healthy controls.

Supplementary Table S5. Summary of single-cell TCR sequencing.

Supplementary Table S6. HLA genotyping and MHC-peptide binding prediction.

Supplementary Table S7. Antigen specificity TCR clustering and potential PD-specific TCR groups.

References

1. Schmiedel, B.J., et al. Impact of Genetic Polymorphisms on Human Immune Cell Gene Expression. *Cell*. **175**, 1701-1715.e16 (2018).
2. Monaco, G., et al. RNA-Seq Signatures Normalized by mRNA Abundance Allow Absolute Deconvolution of Human Immune Cell Types. *Cell Rep*. **26**, 1627-1640.e7 (2019).
3. Mabbott, N.A., et al. An expression atlas of human primary cells: inference of gene function from coexpression networks. *BMC Genomics*. **14**, 632 (2013).
4. Martens, J.H.A. and H.G. Stunnenberg. BLUEPRINT: mapping human blood cell epigenomes. *Haematologica*. **98**, 1487-1489 (2013).
5. Novershtern, N., et al. Densely interconnected transcriptional circuits control cell states in human hematopoiesis. *Cell*. **144**, 296-309 (2011).
